# Functional and Genomic Analysis of *Leuconostoc citreum* DMLC16 Reveals Its Potential as a Probiotic and Antimicrobial Starter

**DOI:** 10.4014/jmb.2507.07004

**Published:** 2025-09-11

**Authors:** Sumin Lee, Minkyeong Kim, Sojeong Heo, Yura Moon, Gawon Lee, Do-Won Jeong

**Affiliations:** Department of Food and Nutrition, Dongduk Women’s University, Seoul 02748, Republic of Korea

**Keywords:** *Leuconostoc citreum*, functional property, antibacterial activity, antifungal activity, genome, starter

## Abstract

This study aimed to assess the probiotic potential of *Leuconostoc citreum* DMLC16 previously selected as a fermentation starter candidate based on safety and enzymatic activity evaluations by further examining its health-promoting properties and antimicrobial efficacy. Strain DMLC16 exhibited higher acid tolerance, bile salt resistance, and intestinal adhesion ability than its type strain *L. citreum* KACC 11860^T^. Moreover, it demonstrated antimicrobial activities against a range of foodborne pathogens and spoilage organisms, including *Bacillus cereus*, *Enterococcus faecalis*, *Listeria monocytogenes*, *Staphylococcus aureus*, *Alcaligenes xylosoxidans*, *Flavobacterium* sp., and *Salmonella enterica*. Strain DMLC16 also showed antifungal activities against *Clonostachys rosea*, *Epicoccum nigrum*, and *Penicillium citrinum*. Whole-genome analysis confirmed the safety of DMLC16, revealing the absence of toxin-encoding and plasmids containing acquired antibiotic resistance genes. It also identified genes associated with enzymatic function, salt tolerance, and survival under extreme conditions such as gastric acidity. Although no specific bacteriocin genes were detected, genes implicated in the production of antimicrobial substances were present. The complete genome sequence of DMLC16 provides valuable insight into genetic determinants underlying its probiotic traits and supports its potential application as a functional starter culture or probiotic in the food industry.

## Introduction

*Leuconostoc citreum* has been isolated in a variety of habitats, including fermented foods such as artisanal cheese [1], sourdough bread [2], and kimchi [3]. In industrial fermentation, *L. citreum* has been used as a starter culture to produce dairy products and fermented vegetables [3-5]. As a heterofermentative lactic acid bacterium, *L. citreum* plays a significant role in enhancing flavor and texture during food fermentation processes [3-5].

Since 2007, the European Food Safety Authority (EFSA) has included *L. citreum* on its Qualified Presumption of Safety (QPS) list of microorganisms deemed safe for use in food and feed [6]. Furthermore, the International Dairy Federation (IDF) has indicated that *L. citreum* is suitable for use as a fermentation starter in dairy and fish products [7]. In addition, *L. citreum* is listed in the food ingredient database maintained by the Korean Ministry of Food and Drug Safety (MFDS). These findings collectively indicate that *L. citreum* can be safely used in food. Therefore, if strains with superior fermentation or health-promoting functionalities are selected within this species, they can be safely applied to the food and functional food industries without additional safety validation required for species not listed in the QPS, IDF, or the MFDS food ingredient database.

In previous studies, a total of 49 *L. citreum* strains have been isolated from kimchi [8, 9]. To select potential starter candidates for fermented foods, safety assessments and fermentation suitability evaluations, including antimicrobial activity, were conducted for isolated *L. citreum* strains [10]. Through this process, *L. citreum* DMLC16 was identified as a starter candidate, exhibiting sensitivity to seven antibiotics and protease activity under 6% salt concentration conditions [10]. Furthermore, to determine whether strain DMLC16 possesses not only fermentation starter properties, but also probiotic functionalities, *in vitro* functional evaluations along with antibacterial and antifungal activity assays were conducted in this study. Genome analysis was also performed to verify fermentation suitability and probiotic potential of strain DMLC16. Through these results, this study aimed to determine the potential of DMLC16 as a novel fermentation starter strain with possible industrial applications.

## Materials and Methods

### Bacterial Strains and Culture Conditions

*L. citreum* DMLC16, previously isolated from kimchi as a potential starter strain based on safety and fermentation suitability evaluations [10], was used to assess probiotic functionality, antimicrobial activity, and genomic characteristics. The type strain *L. citreum* KACC 11860^T^ (=NRIC 1776) was used as a control. All *Leuconostoc* strains were cultured in de Man–Rogosa–Sharpe (MRS) broth (Becton, Dickinson and Co., USA) at 30°C for 16 h.

### Acid Tolerance

*L. citreum* was cultured in MRS broth until reaching an OD_600_ of approximately 0.7. It was then inoculated at 1%(v/v) into MRS broth adjusted to pH 2.5 using HCl, followed by incubation at 30°C for 3 h. Viable cell counts (CFU/ml) were then determined. For the control, *L. citreum* was also inoculated into MRS broth (pH 6.5) without pH adjustment under the same conditions followed by measurements of viable cell counts. Acid tolerance was assessed by comparing the survival rate in the pH 2.5 medium relative to that of the control. All experiments were performed in triplicate.

### Bile Salt Tolerance

Following the same procedure as for acid tolerance, *L. citreum* was cultured in MRS broth until reaching an OD_600_ of approximately 0.7. It was then inoculated at 1% (v/v) into MRS broth supplemented with 0.3% (w/v) oxgall (MB Cell, Republic of Korea) followed by incubation at 30°C for 6 h. Viable cell counts (CFU/ml) were subsequently determined. As a control, *L. citreum* was also inoculated into MRS broth without oxgall supplementation under the same conditions followed by measurements of viable cell counts. Bile salt tolerance was assessed by comparing the survival rate in the oxgall-supplemented medium relative to that of the control. All experiments were performed in triplicate.

### Intestinal Adhesion Ability

The intestinal adhesion ability was evaluated using a mucin-binding assay as described by Azcarate-Peril *et al*.(2009) with slight modifications [11]. Briefly, a 96-well plate was coated with 100 μl of mucin from porcine stomach (10 mg/ml; Sigma-Aldrich, USA) and incubated at 4°C for 24 h. Wells were then washed twice with 0.85%(w/v) NaCl solution. *L. citreum* cells cultured in MRS broth to an OD_600_ of approximately 0.7 were harvested and 100 μl of the culture was added to each mucin-coated well. The plate was incubated at 30°C for 2 h. After incubation, wells were centrifuged at 4,000 rpm for 2 min to remove non-adherent cells and the supernatant was discarded. Remaining wells were washed five times with 0.85% (w/v) NaCl to eliminate loosely bound cells. Subsequently, 0.1% (w/v) Triton X-100 (Sigma-Aldrich) was added to each well to detach adherent cells. Recovered cells were serially diluted in 0.85% NaCl and plated onto MRS agar (Becton, Dickinson and Co.). Colony counts were determined after incubation at 30°C for 48 h. The adhesion rate was defined as the ratio of viable cell counts recovered from mucin-coated wells to viable cell counts from MRS broth (blank). All experiments were performed in triplicate.

### Antibacterial Activity

Antibacterial activities of *L. citreum* DMLC16 against nine spoilage or foodborne pathogenic bacteria (*Bacillus cereus* KCCM 11341, *Enterococcus faecalis* KCTC 2011, *Listeria monocytogenes* ATCC 19111, *Staphylococcus aureus* ATCC 12692, *Alcaligenes xylosoxidans* KCCM 40240, *Escherichia coli* O157:H7 EDL 933, *Flavobacterium* sp. KCCM 11374, *Salmonella enterica* KCCM 11862, and *Vibrio parahaemolyticus* KCTC 2729) were evaluated using the agar well diffusion method [12]. Briefly, indicator pathogens were cultured overnight in Tryptic Soy Broth (TSB; Becton, Dickinson and Co.) and subsequently inoculated into fresh TSB at 1% (v/v) followed by incubation until reaching an OD_600_ of 1.0. Then 200 μl of each culture was spread onto Tryptic Soy Agar (TSA; Becton, Dickinson and Co.) plates. Wells were aseptically punched into the agar Using a sterile 6-mm cork borer. A 20 μl aliquot of the concentrated supernatant from *L. citreum* DMLC16 was added into each well. The concentrated culture supernatant was prepared by culturing DMLC16 in MRS broth at 30°C for 24 h, followed by centrifugation and ten-fold concentration using a HyperVAC centrifugal vacuum concentrator (VC2124, Hanil Scientific Inc., Republic of Korea). Plates were incubated at 30°C for 18 h. Antibacterial activity was assessed by measuring the diameter of the inhibition zones around each well. All experiments were performed independently in triplicate.

### Antifungal Activity

Antifungal activities of *L. citreum* DMLC16 y against food spoilage fungi were evaluated using a dual-culture overlay assay [13]. The indicator fungi used for antifungal activity assessment included nine strains reported to be associated with food spoilage: *Amylomyces rouxii* KCCM 60146, *Aspergillus awamori* KCTC 6902, *Aspergillus oryzae* SNU-G, *Cladosporium herbarum* KACC 47604, *Clonostachys rosea* KACC 40320, *Epicoccum nigrum* KACC 47166, *Penicillium camemberti* KCCM 60340, *Penicillium citrinum* KCCM 60384, and *Rhizopus oryzae* KACC 40256.

To assess antifungal activities, *L. citreum* DMLC16 was first streaked in a 2 cm line onto MRS agar and incubated at 30°C for 48 h. Separately, indicator fungi were cultured on Potato Dextrose Agar (PDA; Becton, Dickinson and Co.) at 30°C for 7 days. Spores were harvested using a 0.1% Tween 80 (Daejung Chemicals & Metals Co. Ltd., Republic of Korea) solution. A soft PDA medium (1% agar) containing 10^4^ spores/ml was then overlaid (10 ml) onto the MRS agar plates where strain DMLC16 had been pre-streaked and incubated. Plates were further incubated at 30°C for an appropriate period depending on the fungal strain. Antifungal activity was evaluated by measuring the clear zone formed around the streaked bacterial line (Table S1). All experiments were performed independently in triplicate.

### Genome Sequencing

Whole-genome sequencing of strain DMLC16 was performed by CJ Bioscience, Inc. (Republic of Korea) using a PacBio Sequel platform (Pacific Bioscience, USA) with a Single-Molecule Real-Time (SMRT) sequencing system (10 Kbp). A total of 41,442 reads were obtained, yielding 778.33× genome coverage. These reads were assembled into a single contig using the HGAP4 algorithm of SMRT Link (version 10.1.0; Pacific Biosciences) on CLC Genomics Workbench ver. 7.5.1 (CLC Bio, Denmark). Genome annotation was performed using the NCBI Prokaryotic Genome Annotation Pipeline (version 4.6) [14]. Open Reading Frames (ORFs) were predicted using Glimmer 3 [15]. They were searched and analyzed against the Clusters of Orthologous Groups (COG) database [15] and the SEED database (accessed on May 21, 2024, at https://rast.nmpdr.org/rast.cgi).

### Statistical Analysis

One-way analysis of variance (ANOVA) followed by Duncan’s multiple range test was used to evaluate significant differences among the mean values of probiotic potential and antimicrobial activity. Differences were considered statistically significant at *p* < 0.05. All statistical analyses were performed using the SPSS software package version 29.0 (SPSS, IBM, USA).

### Nucleotide Sequence Accession Number

The complete genome sequence of *L. citreum* DMLC16 was deposited in the DDBJ/ENA/GenBank under accession number CP151526 and the strain was deposited at the Korean Culture Center of Microorganisms under accession number KFCC12011P.

## Results and Discussion

### Probiotic Properties of *Leuconostoc citreum* DMLC16

**Fig. 1:**
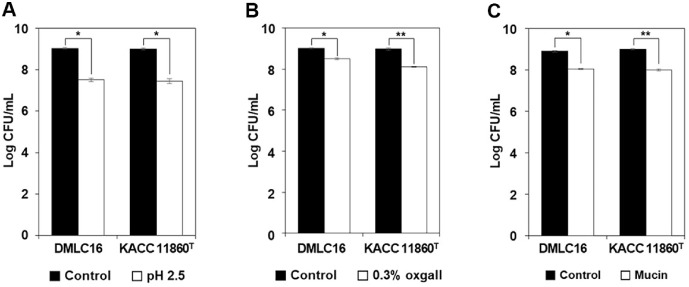
Probiotic potential characterization of *L. citreum* DMLC16 as a starter candidate. (**A**) Acid tolerance, (**B**) Bile salt tolerance, and (**C**) Intestinal attachment ability. Data are expressed as mean ± standard deviation of three experiments. Statistical relevance was analyzed using Duncan’s multiple range test; * indicates *p* < 0.05.

According to the WHO/FAO, probiotics are defined as “live microorganisms which, when administered in adequate amounts, confer a health benefit on the host” [16]. For lactic acid bacteria to be effective as probiotics, they must be able to survive under harsh conditions such as low pH levels (below pH 3) similar to those in the digestive tract and in media containing higher concentrations of bile salts than those found in the intestine. Therefore, acid tolerance and bile salt tolerance of *L. citreum* DMLC16 selected as a starter candidate were evaluated. *L. citreum* DMLC16 showed a viable cell count of 7.50 ± 0.08 log CFU/ml in MRS broth adjusted to pH 2.5 and a viable cell count of 9.03 ± 0.04 log CFU/ml in unadjusted broth (pH 6.5), corresponding to a survival rate of 83.13%. The type strain *L. citreum* KACC 11860^T^ used as a control exhibited a viable cell count of 7.45 ± 0.12 log CFU/ml at pH 2.5 and 9.00 ± 0.05 log CFU/ml in the unadjusted medium, showing a survival rate of 82.78%. This indicated that DMLC16 had a slightly higher acid tolerance than the type strain (Fig. 1A).

Bile salt tolerance was assessed using 0.3% oxgall. Similar to the acid tolerance test, viable cell counts of DMLC16 with and without oxgall supplementation were measured. The viable cell count was 8.50 ± 0.04 log CFU/ml in the presence of 0.3% oxgall and 9.00 ± 0.03 log CFU/ml in the absence of 0.3% oxgall, showing a relative survival rate of 94.35% in the presence of 0.3% oxgall compared to that in the absence of 0.3% oxgall. For the control strain *L. citreum* KACC 11860^T^, the viable count was 8.11 ± 0.02 log CFU/ml in oxgall-supplemented medium and 8.98 ± 0.06 log CFU/ml in the control medium, showing a relative survival rate of 90.30% in the presence of 0.3% oxgall compared to the control. This demonstrated that DMLC16 exhibited a greater bile salt tolerance than the type strain (Fig. 1B). These results indicate that *L. citreum* DMLC16 exhibits greater acid and bile salt tolerance than the type strain.

If strain DMLC16 can survive these harsh conditions and subsequently adhere to intestinal surfaces, its effectiveness as a probiotic would be further enhanced. Therefore, intestinal adhesion ability of strain DMLC16 was evaluated indirectly using mucin [17]. DMLC16 exhibited a viable cell count of 8.05 ± 0.02 log CFU/ml in a mucin-coated medium (10 mg/ml) and a viable count of 8.89 ± 0.03 log CFU/ml in a medium not coated with mucin. Thus, the relative adhesion rate of DMLC16 was approximately 90.48% in the presence of mucin compared to that in the absence of mucin. The control strain *L. citreum* KACC 11860^T^ showed a viable count of 8.00 ± 0.04 log CFU/ml in mucin-coated medium and 8.99 ± 0.02 log CFU/ml in the control medium. Thus, the relative adhesion rate of the control strain was 88.98% in the presence of mucin compared to that in the absence of mucin. Thus, DMLC16 exhibited a slightly better adhesion than the type strain (Fig. 1C). These results suggest that DMLC16 has superior probiotic properties to the reference strain.

The above results demonstrate the *in vitro* probiotic potential of strain DMLC16. Compared to the standard strain, DMLC16 exhibited higher bile salt resistance, acid tolerance, and intestinal adhesion ability. However, it cannot be guaranteed that these results will be replicated in the human body. Therefore, further *in vivo* studies and human trials are necessary to verify its functionality. If its efficacy is confirmed in those studies, DMLC16 could be utilized as a probiotic strain.

### Antibacterial Activity of *L. citreum* Strain DMLC16

A fermentation starter with antibacterial activity can suppress the growth of contaminating or pathogenic bacteria during fermentation, thereby enhancing product safety and quality [18]. Accordingly, antibacterial activities of *L. citreum* DMLC16 against nine spoilage and pathogenic bacterial strains (*B. cereus* KCCM 11341, *E. faecalis* KCTC 2011, *L. monocytogenes* ATCC 19111, *S. aureus* ATCC 12692, *A. xylosoxidans* KCCM 40240, *E. coli* O157:H7 EDL 933, *Flavobacterium* sp. KCCM 11374, *S. enterica* KCCM 11862, and *V. parahaemolyticus* KCTC 2729) were evaluated (Table 1). As a negative control, the medium was adjusted to pH 4.5, corresponding to the pH of the culture supernatant of strain DMLC16, and its antibacterial activity was examined. It was confirmed that no inhibition was observed on the growth of the indicator strain, indicating that the effect was not due to the culture pH. Results showed that DMLC16 did not exhibit antibacterial activity against *E. coli* O157:H7 EDL 933 or *V. parahaemolyticus* KCTC 2729. However, it showed antibacterial activities against the other seven strains. Compared to the control strain *L. citreum* KACC 11860^T^, DMLC16 demonstrated stronger antibacterial activities against *L. monocytogenes* ATCC 19111, *S. aureus* ATCC 12692, and *Flavobacterium* sp. KCCM 11374.

### Antifungal Activity of *L. citreum* Strain DMLC16

Fungal contamination during food production is a major issue that can lead to quality deterioration, reduced shelf life, and food safety concerns. Foods such as bakery products, dairy items, and processed meats manufactured in humid environments are particularly vulnerable to fungal growth. Some fungi can produce mycotoxins that pose health risks to consumers [19]. To address these challenges, antifungal probiotics are emerging as eco-friendly alternatives. Certain lactic acid bacteria (*e.g.*, *Lactobacillus*, *Bifidobacterium*) can inhibit fungal growth by producing lactic acid, acetic acid, and antimicrobial peptides and by lowering the pH to create unfavorable conditions for fungal proliferation [20]. Consequently, antifungal probiotics are gaining attention in the food industry as a way to minimize the use of chemical preservatives while maintaining product freshness and safety. Moreover, when probiotics exhibit antifungal activities, they might further enhance human health by inhibiting the growth of pathogenic fungi, thereby expanding functional benefits of probiotics. As a result, research on antifungal probiotics has been steadily increasing [21, 22].

To evaluate the antifungal potential of strain DMLC16, its antifungal activities against nine fungal species known as spoilage or pathogenic fungi in foods were tested (Table S1). Strain DMLC16 exhibited antifungal activities against *Clonostachys rosea*, *Epicoccum nigrum*, and *Penicillium citrinum* (Table 1). Specifically, DMLC16 formed clear zones measuring 3.40 ± 0.05 mm, 10.10 ± 0.37 mm, and 1.20 ± 0.00 mm against *C. rosea*, *E. nigrum*, and *P. citrinum*, respectively. At the significance level of *p* < 0.05, strain DMLC16 exhibited significantly higher antifungal activity against *C. rosea* compared to the reference strain *L. citreum* KACC 11860^T^, but showed significantly lower activity against *P. citrinum*.

*C. rosea* and *E. nigrum* are known as spoilage fungi of soybean crops and other foods [23-25]. Although their pathogenicity to humans has not been reported, their presence in food production and storage environments suggests that they could contribute to spoilage. In contrast, *P. citrinum* is frequently detected in spoiled foods. It is known to produce citrinin, a mycotoxin associated with nephrotoxicity [26]. Although not all *P. citrinum* strains are necessarily harmful, the potential risk they pose means that foods contaminated by them should be avoided. From this perspective, inhibitory activities of *L. citreum* against these three fungi suggest its potential to reduce fungal contamination in foods. However, relatively small differences observed among strains suggest that its antifungal activity might be characteristic of the species rather than specific to the strain.

### General Genome Properties of *L. citreum* Strain DMLC16

In a previous study, *L. citreum* strain DMLC16 exhibited protease activity at a 6% salt concentration and demonstrated acid production at a 3% salt concentration [10]. Its ability to survive at 6% salt concentration suggests that it possesses potential to degrade proteins in fermented foods with approximately 6% salinity and potential to produce organic acids under 3% salt conditions. These properties imply that, when used as a starter culture for fermented foods, this strain could enhance sensory characteristics of the products by generating amino acids and organic acids.

In the present study, *L. citreum* strain DMLC16 demonstrated superior tolerance to bile salts and acidic conditions. It also possessed enhanced intestinal adhesion ability compared to the control strain, thereby confirming its potential as a probiotic (Fig. 1). Moreover, strain DMLC16 exhibited antimicrobial and antifungal activities against spoilage and pathogenic microorganisms. This indicates that it possesses additional functional attributes that can further support its potential as both a fermentation starter and a probiotic (Table 1). Accordingly, genomic analysis was conducted to elucidate the genetic basis underlying the fermentation, probiotic, and antimicrobial capabilities of *L. citreum* strain DMLC16. The complete genome of *L. citreum* DMLC16 consists of a circular chromosome of 1,876,553 bp with a G+C content of 38.97 mol%, and no plasmids were detected. Seventy-one tRNA genes and 12 rRNA genes were identified in its genome. Genomic analysis predicted 1,931 open reading frames, of which 1,668 genes were assigned functionally to categories based on the COG database. Excluding the category “function unknown”, the category of “carbohydrate transport and metabolism” (147 genes, 8.8%) was the most abundant COG category, followed by “translation, ribosomal structure and biogenesis” (140 genes, 8.4%) and “transcription” (127 genes, 7.6%). The SEED subsystem categorized 748 genes. Protein metabolism (123 genes, 16.4%) was the most abundant subsystem category, followed by carbohydrates (103 genes, 13.8%).

### Safety Properties Based on Genome of *L. citreum* Strain DMLC16

For microorganisms to be used in food, safety requires the absence of acquired antibiotic resistance and toxic factors such as hemolytic activity [6]. Similarly, for probiotics, the absence of transferable antibiotic resistance genes and toxic factors such as hemolysins is a critical safety criterion [16]. In a previous study, strain DMLC16 was assessed for antibiotic resistance against eight antibiotics following the EFSA guidelines [10]. This strain was found to be sensitive to seven antibiotics (ampicillin, chloramphenicol, clindamycin, erythromycin, gentamicin, streptomycin, and tetracycline) with the exception of kanamycin. As all strains of *Leuconostoc* spp. tested previously exceeded the EFSA cutoff value for kanamycin resistance, the kanamycin resistance observed was inferred to be species-specific rather than strain-specific. Based on these results, a genomic analysis of strain DMLC16 was conducted to identify genetic factors contributing to antibiotic resistance gene including kanamycin resistance gene. Using ResFinder to analyze the genome, genes associated with resistance to kanamycin or other antibiotics were not detected (Table S2).

Further analysis using the Comprehensive Antibiotic Resistance Database (CARD) algorithms, which could predict antibiotic resistance based on protein homology models, identified three genes potentially related to antibiotic resistance (Table 2). However, the homology of these genes was low, ranging from 32.88% to 49.52%. Moreover, BLASTP analysis of deduced amino acid sequences revealed that AADZ99_RS01080, AADZ99_RS05635, and AADZ99_RS08290 corresponded to an alanine racemase, a DMT family transporter, and an M15 family metallopeptidase, respectively, suggesting a limited association with antibiotic resistance. Genome analysis using "kanamycin" as a keyword did not detect any related genes either. Thus, no genes could be clearly identified as responsible for kanamycin resistance in the genome of strain DMLC16. Nevertheless, the minimum inhibitory concentration of kanamycin for DMLC16 was 256 mg/l, which exceeded the EFSA cutoff value for *Leuconostoc* spp. (16 mg/l). Thus, further research is recommended to elucidate the genetic basis for this resistance.

To evaluate the presence of toxic factors related to hemolysis, a genome-wide search using “toxin” and “hemolysin” as keywords was performed. Relevant genes were not detected. Based on these results, strain DMLC16 could be considered as genetically safe as no acquired antibiotic resistance genes or toxic factors were identified.

### Technological Property Based on the Genome of *L. citreum* Strain DMLC16

In a previous study, when strain DMLC16 was used as a fermentation starter, it exhibited growth in a medium containing 6% salt and demonstrated protease activity [10]. Additionally, it produced acid under 3% salt conditions. To investigate its functional characteristics, genes of strain DMLC16 were analyzed. The genome of DMLC16 was found to contain a total of 33 protease genes (Table 3). The absence of amylase and lipase activities observed phenotypically was consistent with its genomic data, which revealed no corresponding enzyme genes.

**Fig. 2:**
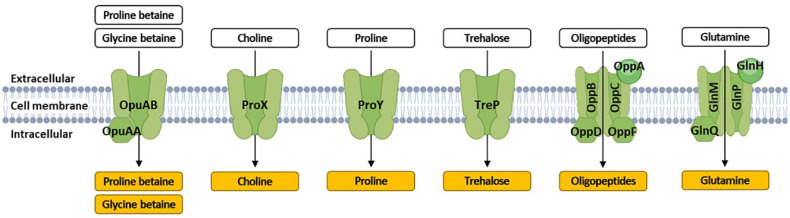
Predicted mechanism of salt-tolerance of *L. citreum* DMLC16 based genomic analysis. Compatible solutes are depicted in boxes. The transporter carrying compatible solutes is indicated by black letters.

To verify the strain's salt tolerance, a genomic analysis was conducted. It is known that in the presence of high external salt concentrations, microorganisms can accumulate osmoprotectants such as choline, glycine betaine, proline betaine, and trehalose within cells to support growth [27, 28]. In line with this, the genome of DMLC16 was found to possess genes encoding transporters responsible for the uptake of osmoprotectants (Fig. 2 and Table S3). These genes were organized into operons, each predicted to encode specific osmoprotectant transport systems (Fig. S1). Additionally, genes encoding membrane proteins with binding functions for choline and betaine (AADZ99_RS00545, AADZ99_RS07110, AADZ99_RS07115, AADZ99_RS07675, AADZ99_RS07850, and AADZ99_RS08270) were identified in the genome of *L. citreum* strain DMLC16.

**Fig. 3:**
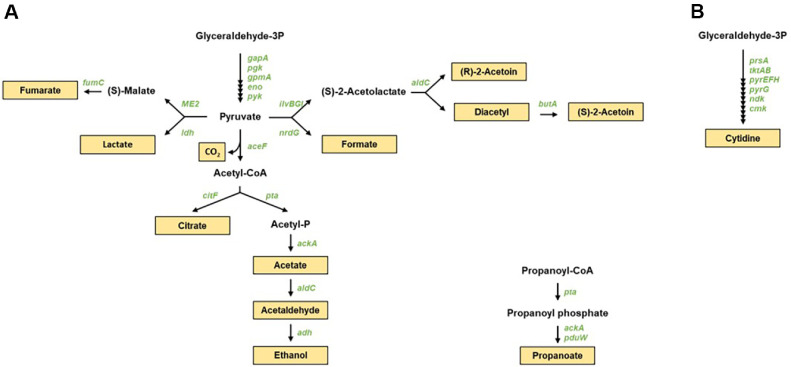
Predicted (A) Organic acid derivatization (B) Cytidine pathway pathways of *L. citreum* DMLC16. Genes and products depicted in green italics and letters in light yellow boxes, respectively. Black arrows correspond to potential enzymatic reactions catalyzed by gene products encoded in the DMLC16 genome.

To analyze acid production capacity, calcium carbonate was used as a substrate, taking advantage of its property of being decomposed upon reaction with acid. Since strain DMLC16 exhibited acid production, a genome analysis was conducted with a focus on organic acid production. The analysis revealed that DMLC16 possessed all genes necessary for the syntheses of acetaldehyde, acetate, acetoin, citrate, diacetyl, ethanol, formate, fumarate, and lactate from glyceraldehyde-3-phosphate, an intermediate generated through glucose metabolism (Fig. 3A and Table S4). The genomic analysis provided supporting evidence for the fermentation suitability of strain DMLC16, allowing experimental findings to be interpreted at the genomic level.

### Probiotic Properties Based on the Genome of *L. citreum* Strain DMLC16

**Fig. 4:**
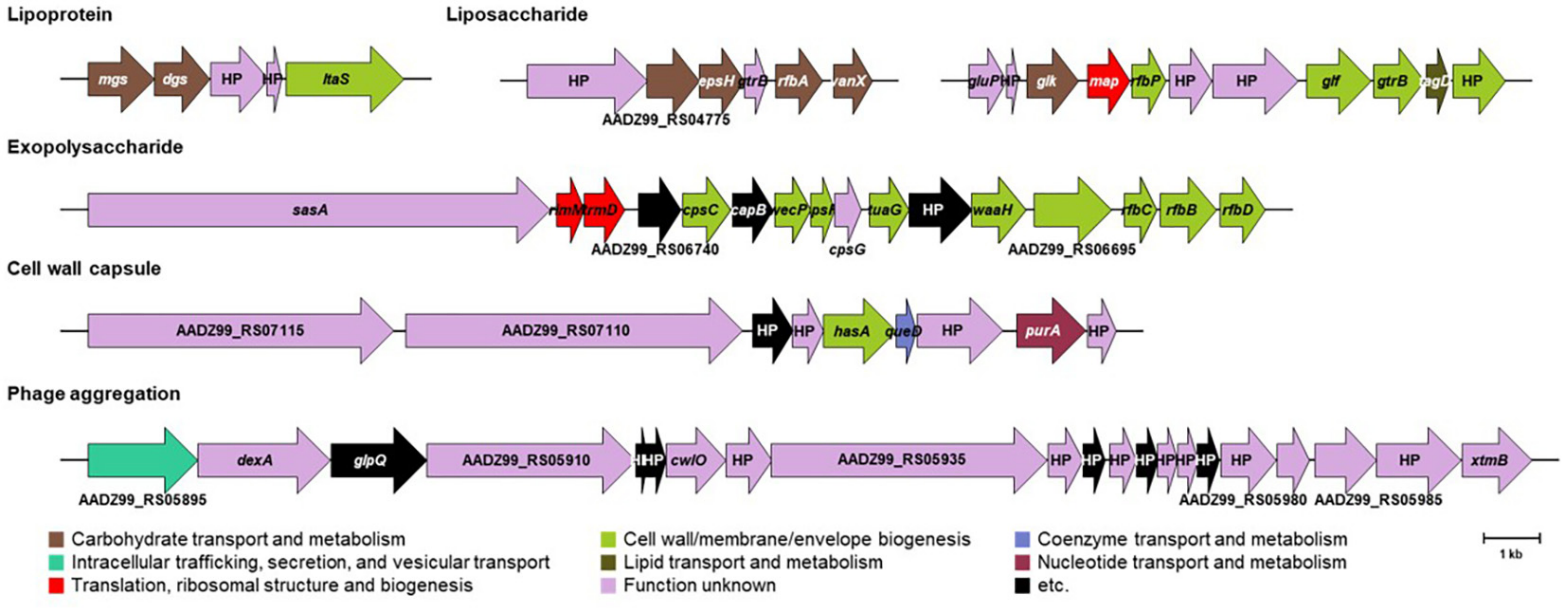
Annotated genes related to probiotic properties of strain DMLC16. The position and orientation of the coding regions are represented by arrows. The name of the arrow is the annotated gene name or the locus number. HP, hypothetical protein.

Probiotics are defined as live microorganisms that confer health benefits to humans and animals [29]. Generally, to contribute to gut health, probiotics must survive harsh conditions of the gastrointestinal tract, including low pH and the presence of bile salts [30]. Bile salt hydrolase (EC 3.5.1.24; choloylglycine hydrolase) is known to catalyze the conversion of conjugated bile salts into free bile salts, thereby enhancing the survivability of probiotics in the gastric environment [31]. The genome of strain DMLC16 encodes a bile salt hydrolase gene (AADZ99_RS03405). Capsule formation also plays a crucial role in enabling microorganisms to withstand harsh environmental conditions [32]. The genome of strain DMLC16 harbors an operon involved in capsule formation, including a hyaluronan synthase gene (AADZ99_RS07080) (Fig. 4 and Table S5). In addition to providing resistance to external stressors, the capsule can enhance the strain’s adhesion ability. Bacterial adhesion is a key trait that allows probiotics to establish residency within the gut [33]. Exopolysaccharides (EPS), liposaccharides, and extracellular glucans are known to contribute to adhesion to the human intestinal mucosa [34]. Notably, the genome of DMLC16 was found to possess operons responsible for the production of EPS, liposaccharides, and extracellular glucans (Fig. 4 and Table S5).

### Antimicrobial Properties Based on the Genome of *L. citreum* Strain DMLC16

Resistance against spoilage and foodborne pathogens within fermented foods is essential for maintaining product quality and ensuring food safety. Similarly, probiotics must be able to decrease pathogenic bacteria in the gut after colonization. Strain DMLC16 demonstrated both antibacterial and antifungal activities (Table 1). Since genome analysis did not detect genes responsible for bacteriocin production, other antimicrobial factors such as organic acids and volatile substances were investigated. The analysis revealed that DMLC16 harbored genes involved in the production of organic acids, including lactic acid, acetic acid, formic acid, and propionic acid (Fig. 3 and Table S4). Furthermore, genes related to the syntheses of volatile compounds such as ethanol, acetoin, and diacetyl, as well as other antimicrobial substances such as phenylacetic acid, cytidine, CO_2_, and acetaldehyde were identified. These findings suggest that the antimicrobial activity of strain DMLC16 might be attributed to its production of these organic acids and volatile compounds.

Consequently, the strain *L. citreum* DMLC16 isolated from kimchi was selected as a potential starter candidate based on its antibiotic susceptibility, absence of hemolytic activity, technological and functional properties for fermentation and health, and antimicrobial activity. Genome analysis revealed that *L. citreum* DMLC16 did not possess virulence factors or acquired antibiotic resistance genes. Additionally, the genome of strain DMLC16 meets the criteria required for a promising probiotic and starter culture candidate. The complete genome sequence of *L. citreum* DMLC16 provides a genetic foundation for future comparative and functional genomic studies. It supports the development of this strain for applications in food, animal feed, and medical industries.

## Supplemental Materials

Supplementary data for this paper are available on-line only at http://jmb.or.kr.



## Figures and Tables

**Table 1:** Antibacterial and antifungal activities of *L. citreum* strain DMLC16.

Species	Strain	Clear zone size (mm)	
		DMLC16	KACC 11860^T^
Gram-positive bacteria			
*Bacillus cereus*	KCCM 11341	1.25 ± 0.35	1.25 ± 0.42
*Enterococcus faecalis*	KCTC 2011	1.50 ± 0.71	2.50 ± 0.42*
*Listeria monocytogenes*	ATCC 19111	3.25 ± 0.14*	3.00 ± 0.07
*Staphylococcus aureus*	ATCC 12692	1.75 ± 0.42*	0.75 ± 0.14
Gram-negative bacteria			
*Alcaligenes xylosoxidans*	KCCM 40240	2.75 ± 0.49	2.25 ± 0.14
*Flavobacterium* sp.	KCCM 11374	1.00 ± 0.28*	0.75 ± 0.35
*Salmonella enterica*	KCCM 11862	1.50 ± 0.71	1.75 ± 0.35*
Fungi			
*Clonostachys rosea*	KACC 40320	3.40 ± 0.05*	3.00 ± 0.05
*Epicoccum nigrum*	KACC 47166	10.10 ± 0.37	10.10 ± 0.40
*Penicillium citrinum*	KCCM 60384	1.20 ± 0.00	2.10 ± 0.01*

Data are expressed as mean ± standard deviation of three experiments. Asterisk in a column indicate significant differences at *p* < 0.05 by Duncan’s multiple range test.

**Table 2:** Prediction of antibiotic resistance related gene for *L. citreum* DMLC16 by CARD.

Antimicrobial resistance gene Family	Drug class	Identity of matching region (%)	EC No.	Annotated gene function by BLAST P	Locus Tag
Glycopeptide resistance gene cluster, vanT	Glycopeptide antibiotic	33.07	5.1.1.1	Alanine racemase	AADZ99_RS01080
Small multidrug resistance antibiotic efflux pump	Disinfecting agents and antiseptics	49.52	–	DMT family transporter	AADZ99_RS05635
vanY, Glycopeptide resistance gene cluster	Glycopeptide antibiotic	32.88	3.4.17.14	M15 family metallopeptidase	AADZ99_RS08290

Enzyme Commission (EC) number is a numerical classification scheme for enzymes based on chemical reactions they catalyze. EC numbers are based on genes of strain DMLC16. Identity of matching region (%) means the amino acid sequence homology between two strains by CARD analysis. Abbreviation: –, not identified.

**Table 3:** Putative protease genes identified in the genome of *L. citreum* DMLC16.

Gene product	EC No.	Gene locus
Aminopeptidase	3.4.11.-	AADZ99_RS08275
ATP-dependent zinc metalloprotease FtsH	3.4.24.-	AADZ99_RS07880
D-Ala-D-Ala dipeptidase	3.4.13.22	AADZ99_RS04795
Dipeptidase E	3.4.13.21	AADZ99_RS05690
Endopeptidase Clp	3.4.21.92	AADZ99_RS02030
Endopeptidase Clp	3.4.21.92	AADZ99_RS05440
Glutamyl aminopeptidase	3.4.11.7	AADZ99_RS02550
Group B oligopeptidase PepB	3.4.24.-	AADZ99_RS01830
Hypothetical protein	3.4.-.-	AADZ99_RS01115
Hypothetical protein	3.4.-.-	AADZ99_RS05400
Hypothetical protein	3.4.-.-	AADZ99_RS05925
Hypothetical protein	3.4.24.-	AADZ99_RS01385
Lipoprotein NlpD/LppB like protein	3.4.-.-	AADZ99_RS01120
Membrane alanyl aminopeptidase	3.4.11.2	AADZ99_RS02310
Methionyl aminopeptidase	3.4.11.18	AADZ99_RS06430
Muramoyltetrapeptide carboxypeptidase	3.4.17.13	AADZ99_RS04335
Peptidase Do	3.4.21.107	AADZ99_RS00835
Peptidase Do	3.4.21.107	AADZ99_RS08180
Probable endopeptidase p60	3.4.-.-	AADZ99_RS01110
Probable inactive metalloprotease YmfF	3.4.24.-	AADZ99_RS07025
Protease HtpX like protein	3.4.24.-	AADZ99_RS03055
Putative zinc metalloprotease	3.4.24.-	AADZ99_RS03285
Putative zinc metalloproteinase in scaA 5'region	3.4.24.-	AADZ99_RS00645
Rhomboid protease	3.4.21.105	AADZ99_RS06445
Serine-type D-Ala-D-Ala carboxypeptidase	3.4.16.4	AADZ99_RS01250
Signal peptidase I	3.4.21.89	AADZ99_RS05190
Tripeptide aminopeptidase	3.4.11.4	AADZ99_RS04915
Type 4 prepilin-like protein leader peptide-processing enzyme	3.4.23.43, 2.1.1.-	AADZ99_RS08820
Uncharacterized protein	3.4.21.-	AADZ99_RS02575
Xaa-Pro aminopeptidase	3.4.11.9	AADZ99_RS05325
Xaa-Pro dipeptidase	3.4.13.9	AADZ99_RS02395
Xaa-Pro dipeptidase	3.4.13.9	AADZ99_RS08435
Zinc D-Ala-D-Ala carboxypeptidase	3.4.17.14	AADZ99_RS08290

Enzyme Commission (EC) number is a numerical classification scheme for enzymes based on chemical reactions they catalyze.
