# Supplementary material for: Functional and Genomic Analysis of Leuconostoc citreum DMLC16 Reveals Its Potential as a Probiotic and Antimicrobial Starter
Source: J Microbiol Biotechnol. 2025 Sep 11;35:e2507004. doi: 10.4014/jmb.2507.07004 (PMC12438955; doi:10.4014/jmb.2507.07004)
Supplement: Supplementary file 1 [file jmb-35-e2507004-supple.pdf]

J. Microbiol. Biotechnol. 2025. 35: e2507004
https://doi.org/10.4014/jmb.2507.07004

Supplementary Figure and Tables

Functional and genomic analysis of *Leuconostoc citreum* DMLC16 reveals its potential as a probiotic and antimicrobial starter

Sumin Lee, Sojeong Heo, Yura Moon, Minkyeong Kim, Gawon Lee, and Do-Won Jeong*

Department of Food and Nutrition, Dongduk Women's University, Seoul 02748, Republic of Korea

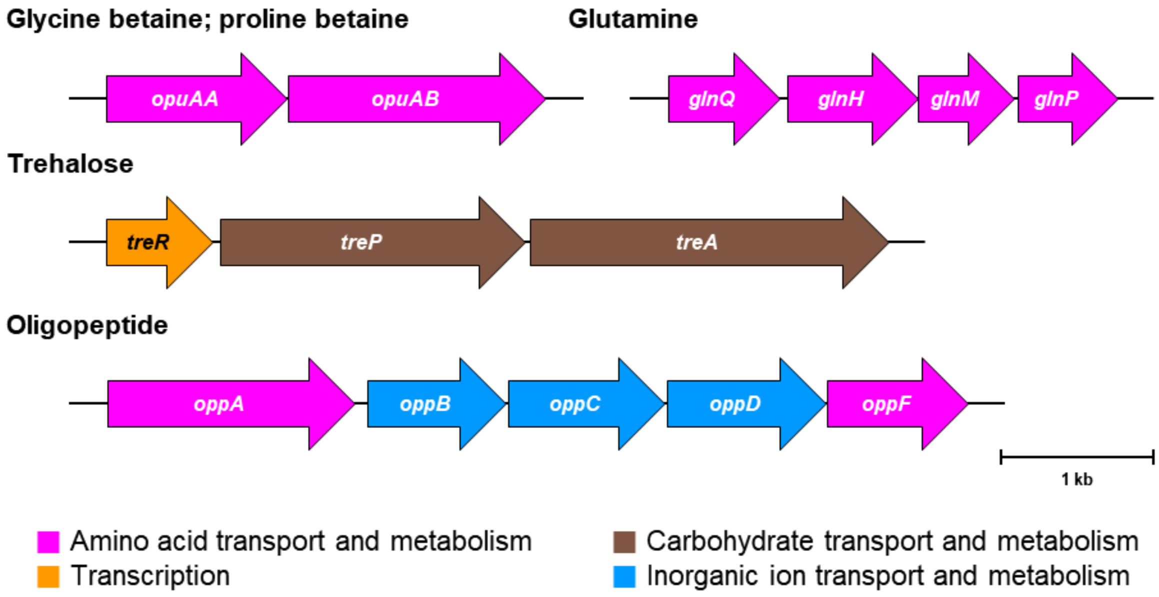

Fig. S1. Genetic structure involved in the uptake of osmoprotectants in *Leuconostoc citreum* DMLC16. The position and orientation of the coding regions are represented by arrows.

Table S1. List of fungi used for antifungal activity and incubation time.

Species	Strain	Incubation time
Amylomyces rouxii	KCCM 60146	48 hr
Aspergillus awamori	KCTC 6902	48 hr
Aspergillus oryzae	SNU-G	48 hr
Cladosporium herbarum	KACC 47604	48 hr
Clonostachys rosea	KACC 40320	72 hr
Epicoccum nigrum	KACC 47166	120 hr
Penicillium camemberti	KCCM 60340	48 hr
Penicillium citrinum	KCCM 60384	72 hr
Rhizopus oryzae	KACC 40256	48 hr

Table S2. Prediction of antibiotic resistance related gene for *Leuconostoc citreum* DMLC16 by ResFinder

Class	Antimicrobial	WGS-predicted phenotype
Aminocyclitol	Spectinomycin	No resistance
Aminoglycoside	Amikacin	No resistance
	Apramycin	No resistance
	Arbekacin	No resistance
	Astromicin	No resistance
	Butiromycin	No resistance
	Butirosin	No resistance
	Dibekacin	No resistance
	Fortimicin	No resistance
	Gentamicin	No resistance
	Hygromycin	No resistance
	Isepamicin	No resistance
	Kanamycin	No resistance
	Kasugamycin	No resistance
	Lividomycin	No resistance
	Neomycin	No resistance
	Netilmicin	No resistance
	Paromomycin	No resistance
	Ribostamycin	No resistance
	Sisomicin	No resistance
	Streptomycin	No resistance
	Tobramycin	No resistance
	Unknown aminoglycoside	No resistance
Amphenicol	Chloramphenicol	No resistance
	Florfenicol	No resistance
Beta-lactam	Amoxicillin	No resistance
	Amoxicillin+Clavulanic acid	No resistance
	Ampicillin	No resistance
	Ampicillin+Clavulanic acid	No resistance
	Aztreonam	No resistance
	Cefepime	No resistance
	Cefixime	No resistance
	Cefotaxime	No resistance
	Cefotaxime+Clavulanic acid	No resistance
	Cefoxitin	No resistance
	Ceftazidime	No resistance
	Ceftazidime+Avibactam	No resistance
	Ceftriaxone	No resistance
	Cephalothin	No resistance
	Cephalotin	No resistance
	Ertapenem	No resistance
	Imipenem	No resistance
	Meropenem	No resistance
	Penicillin	No resistance
	Piperacillin	No resistance
	Piperacillin+clavulanic acid	No resistance
	Piperacillin+tazobactam	No resistance
	Temocillin	No resistance
	Ticarcillin	No resistance
	Ticarcillin+Clavulanic acid	No resistance
	Unknown beta-lactam	No resistance
Folate pathway antagonist	Sulfamethoxazole	No resistance
	Trimethoprim	No resistance
Fosfomycin	Fosfomycin	No resistance
Glycopeptide	Bleomycin	No resistance
	Teicoplanin	No resistance
	Vancomycin	No resistance
Ionophores	Maduramicin	No resistance

Class	Antimicrobial	WGS-predicted phenotype
	Narasin	No resistance
	Salinomycin	No resistance
Lincosamide	Clindamycin	No resistance
	Lincomycin	No resistance
Macrolide	Azithromycin	No resistance
	Carbomycin	No resistance
	Erythromycin	No resistance
	Oleandomycin	No resistance
	Spiramycin	No resistance
	Telithromycin	No resistance
	Tylosin	No resistance
Nitroimidazole	Metronidazole	No resistance
Oxazolidinone	Linezolid	No resistance
Pleuromutilin	Tiamulin	No resistance
Polymyxin	Colistin	No resistance
Pseudomonic acid	Mupirocin	No resistance
Quinolone	Ciprofloxacin	No resistance
	Fluoroquinolone	No resistance
	Nalidixic acid	No resistance
	Unknown quinolone	No resistance
Rifamycin	Rifampicin	No resistance
Steroid antibacterial	Fusidic acid	No resistance
Streptogramin A	Dalfopristin	No resistance
	Pristinamyciniia	No resistance
	Quinupristin+Dalfopristin	No resistance
	Virginiamycinm	No resistance
Streptogramin B	Pristinamycinia	No resistance
	Quinupristin	No resistance
	Virginiamycins	No resistance
Tetracycline	Doxycycline	No resistance
	Minocycline	No resistance
	Tetracycline	No resistance
	Tigecycline	No resistance
Under development	Ceftiofur	No resistance

Table S3. Predicted mechanism of salt-tolerance of *Leuconostoc citreum* DMLC16 based genomic analysis

Product	Gene	EC No.	Locus tag
Transporter			
Choline transport system permease protein	*opuAB*		AADZ99_RS07290
Quaternary-amine-transporting ATPase	*opuAA*	3.6.3.32	AADZ99_RS07295
Choline transport system permease protein	*proX*		AADZ99_RS02375
Proline-specific permease	*proY*		AADZ99_RS02950
Trehalose 6-phosphate phosphorylase	*treA*	2.4.1.216	AADZ99_RS07755
Glucose-specific phosphotransferase enzyme IIA component	*treP*		AADZ99_RS07760
Trehalose operon transcriptional repressor	*treR*		AADZ99_RS07765
Glutathione-binding protein	*oppA*		AADZ99_RS00620
Oligopeptide transport system permease protein	*oppB*		AADZ99_RS00625
Dipeptide transport system permease protein	*oppC*		AADZ99_RS00630
Lipoprotein-releasing system ATP-binding protein	*oppD*		AADZ99_RS00635
Oligopeptide transport ATP-binding protein	*oppF*		AADZ99_RS00640
Polar-amino-acid-transporting ATPase	*glnQ*	3.6.3.21	AADZ99_RS00600
ABC transporter glutamine-binding protein	*glnH*		AADZ99_RS00605
Probable glutamine ABC transporter permease protein	*glnM*		AADZ99_RS00610
Probable glutamine ABC transporter permease protein	*glnP*		AADZ99_RS00615
Membrane protein			
Glucosyltransferase-S			AADZ99_RS00545
Dextransucrase			AADZ99_RS07110
Dextransucrase			AADZ99_RS07115
Dextransucrase			AADZ99_RS07675
Hypothetical protein			AADZ99_RS07850
Autolytic lysozyme			AADZ99_RS08270

Table S4. Prediction of technological property and fermentation properties related gene for *Leuconostoc citreum* DMLC16

Product	Gene	EC No.	Locus tag
Organic acid derivatization			
Glyceraldehyde-3-phosphate dehydrogenase (phosphorylating)	*gapA*	1.2.1.12	AADZ99_RS01450
Phosphoglycerate kinase	*pgk*	2.7.2.3	AADZ99_RS08945
Phosphoglycerate mutase (2,3-diphosphoglycerate-dependent)	*gpmA*	5.4.2.11	AADZ99_RS02545
Phosphoglycerate mutase (2,3-diphosphoglycerate-dependent)	*gpmA*	5.4.2.11	AADZ99_RS08540
Phosphopyruvate hydratase	*eno*	4.2.1.11	AADZ99_RS08490
Dihydrolipoyllysine-residue acetyltransferase	*aceF*	2.3.1.12	AADZ99_RS02240
Phosphate acetyltransferase	*pta*	2.3.1.8	AADZ99_RS02890
Citrate CoA-transferase	*citF*	2.8.3.10	AADZ99_RS00435
Acetate kinase	*ackA*	2.7.2.1	AADZ99_RS01305
Acetolactate decarboxylase	*aldC*	4.1.1.5	AADZ99_RS02410
Alcohol dehydrogenase	*adh*	1.1.1.1	AADZ99_RS00700
Malate dehydrogenase (oxaloacetate-decarboxylating)	*ME2*	1.1.1.38	AADZ99_RS00415
Malate dehydrogenase (oxaloacetate-decarboxylating)	*ME2*	1.1.1.38	AADZ99_RS04205
L-lactate dehydrogenase	*Idh*	1.1.1.27	AADZ99_RS09175
Fumarate hydratase	*fumC*	4.2.1.2	AADZ99_RS00665
Fumarate hydratase	*fumC*	4.2.1.2	AADZ99_RS09125
Acetolactate synthase	*ilvBGI*	2.2.1.6	AADZ99_RS07370
[Formate-C-acetyltransferase]-activating enzyme	*nrdG*	1.97.1.4	AADZ99_RS07360
Diacetyl reductase ((S)-acetoin forming)	*butA*	1.1.1.-, 1.1.1.76, 1.1.1.304	AADZ99_RS00405
Propionate kinase	*pduW*	2.7.2.15	AADZ99_RS03755
Cytidine pathway			
Ribose-phosphate diphosphokinase	*prsA*	2.7.6.1	AADZ99_RS00810
Ribose-phosphate diphosphokinase	*prsA*	2.7.6.1	AADZ99_RS03335
Transketolase	*tktAB*	2.2.1.1	AADZ99_RS03615
Orotate phosphoribosyltransferase	*pyrE*	2.4.2.10	AADZ99_RS05155
Orotidine-5'-phosphate decarboxylase	*pyrF*	4.1.1.23	AADZ99_RS05160
UMP kinase	*pyrH*	2.7.4.22	AADZ99_RS05115
CTP synthase (glutamine hydrolyzing)	*pyrG*	6.3.4.2	AADZ99_RS07385
Nucleoside-diphosphate kinase	*ndk*	2.7.4.6	AADZ99_RS07185
(d)CMP kinase	*cmk*	2.7.4.25	AADZ99_RS03550
5'-nucleotidase	-	3.1.3.5	AADZ99_RS00240
5'-nucleotidase	-	3.1.3.5	AADZ99_RS05310

Table S4. Annotated genes related to the probiotic properties for *Leuconostoc citreum* DMLC16.

Product	Gene	EC No.	Locus tag
Lipoprotein			
1,2-Diacylglycerol 3-alpha-glucosyltransferase	*mgs*	2.4.1.337	AADZ99_RS01980
Diglucosyl diacylglycerol synthase (1,2-linking)	*dgs*	2.4.1.208	AADZ99_RS01985
Hypothetical protein			AADZ99_RS01990
Hypothetical protein			AADZ99_RS01995
Phosphatidylglycerol--membrane-oligosaccharide Glycerophosphotransferase	*ltaS*	2.7.8.20	AADZ99_RS02000
Liposaccharide			
Hypothetical protein			AADZ99_RS04770
Dolichyl-phosphate beta-D-mannosyltransferase		2.4.1.83	AADZ99_RS04775
Uncharacterized glycosyltransferase	*epsH*	2.4.-.-	AADZ99_RS04780
Hypothetical protein	*gtrB*	2.4.-.-	AADZ99_RS04785
Glucose-1-phosphate thymidylyltransferase	*rfbA*	2.7.7.24	AADZ99_RS04790
D-Ala-D-Ala dipeptidase	*vanX*	3.4.13.22	AADZ99_RS04795
Hypothetical protein			AADZ99_RS06395
Glycerol-3-phosphate cytidylyltransferase	*tagD*	2.7.7.39	AADZ99_RS06400
Bactoprenol glucosyl transferase	*gtrB*	2.4.-.-	AADZ99_RS06405
UDP-galactopyranose mutase	*glf*	5.4.99.9	AADZ99_RS06410
Hypothetical protein			AADZ99_RS06415
Hypothetical protein			AADZ99_RS06420
Undecaprenyl-phosphate galactose phosphotransferase	*rfbP*	2.7.8.6	AADZ99_RS06425
Methionyl aminopeptidase	*map*	3.4.11.18	AADZ99_RS06430
Glucokinase	*glk*	2.7.1.2	AADZ99_RS06435
Hypothetical protein			AADZ99_RS06440
Rhomboid protease	*gluP*	3.4.21.105	AADZ99_RS06445
Exopolysaccharide			
DTDP-4-dehydrorhamnose reductase	*rfbD*	1.1.1.133	AADZ99_RS06680
DTDP-glucose 4,6-dehydratase	*rfbB*	4.2.1.46	AADZ99_RS06685
DTDP-4-dehydrorhamnose 3,5-epimerase	*rfbC*	5.1.3.13	AADZ99_RS06690
Putative O-antigen transporter			AADZ99_RS06695
Uncharacterized glycosyltransferase	*waaH*	2.4.1.-	AADZ99_RS06700
Hypothetical protein			AADZ99_RS06705
Uncharacterized glycosyltransferase	*tuaG*	2.4.-.-	AADZ99_RS06710
Hypothetical protein	*cpsG*		AADZ99_RS06715
N-acetylglucosaminyldiphosphodolichol N-acetylglucosaminyltransferase	*cpsF*	2.4.1.141	AADZ99_RS06720
UDP-N-acetylgalactosamine-undecaprenyl-phosphateN-acetylgalactosaminephosphotransferase	*wecP*	2.7.8.40	AADZ99_RS06725
Putative tyrosine-protein kinase	*capB*		AADZ99_RS06730
Capsular polysaccharide biosynthesis protein	*cpsC*		AADZ99_RS06735
Protein-tyrosine-phosphatase		3.1.3.48	AADZ99_RS06740
TRNA (guanine(37)-N(1))-methyltransferase	*trmD*	2.1.1.228	AADZ99_RS06745
Ribosome maturation factor	*rimM*		AADZ99_RS06750
Glycoprotein	*sasA*		AADZ99_RS09570

Cell wall capsule			
Hypothetical protein			AADZ99_RS07075
Adenylosuccinate synthase	*purA*	6.3.4.4	AADZ99_RS07080
Hypothetical protein			AADZ99_RS07085
6-pyruvoyltetrahydropterin synthase	*queD*	4.2.3.12, 4.1.2.50	AADZ99_RS07090
Hyaluronan synthase	*hasA*	2.4.1.212	AADZ99_RS07095
Hypothetical protein			AADZ99_RS07100
Hypothetical protein			AADZ99_RS07105
Dextransucrase		2.4.1.5	AADZ99_RS07110
Dextransucrase		2.4.1.5	AADZ99_RS07115
Phage aggregation			
Collagen alpha-1(II) chain			AADZ99_RS05895
Dextranase	*dexA*	3.2.1.11	AADZ99_RS05900
Glycerophosphodiester phosphodiesterase	*glpQ*	3.1.4.46	AADZ99_RS05905
Dextransucrase		2.4.1.5	AADZ99_RS05910
Hypothetical protein			AADZ99_RS05915
Hypothetical protein			AADZ99_RS05920
Hypothetical protein	*cwlO*	3.4.-.-	AADZ99_RS05925
Hypothetical protein			AADZ99_RS05930
Tail tape measure protein			AADZ99_RS05935
Hypothetical protein			AADZ99_RS05940
Hypothetical protein			AADZ99_RS05945
Hypothetical protein			AADZ99_RS05950
Hypothetical protein			AADZ99_RS05955
Hypothetical protein			AADZ99_RS05960
Uncharacterized protein			AADZ99_RS05965
Hypothetical protein			AADZ99_RS05970
Hypothetical protein			AADZ99_RS05975
Capsid assembly scaffolding protein			AADZ99_RS05980
Structural protein			AADZ99_RS05985
Hypothetical protein			AADZ99_RS05990
Terminase, large subunit	*xtmB*		AADZ99_RS05995
Adehesin			
Halomucin			AADZ99_RS04645
Clumping factor			AADZ99_RS05600
Androglobin			AADZ99_RS08000
Bile salt hydrolase			
Choloylglycine hydrolase		3.5.1.24	AADZ99_RS03405
Biofilm			
Sortase A	*srtA*	3.4.22.70	AADZ99_RS03355
Sortase A	*srtA*	3.4.22.70	AADZ99_RS04125
Hypothetical protein			AADZ99_RS04130
Cell wall binding			
Dextransucrase		2.4.1.5	AADZ99_RS07675
Fibronectin-binding protein			
Uncharacterized protein			AADZ99_RS06540
Lysin			

4-hydroxy-tetrahydrodipicolinate reductase	*dapB*	1.17.1.8	AADZ99_RS06475
4-hydroxy-tetrahydrodipicolinate synthase	*dapA*	4.3.3.7	AADZ99_RS06480
N-acetyldiaminopimelate deacetylase	*dapL*	3.5.1.47	AADZ99_RS06485
2,3,4,5-tetrahydropyridine-2,6-dicarboxylate N-succinyltransferase	*dapD*	2.3.1.117	AADZ99_RS06425
Diaminopimelate decarboxylase	*lysA*	4.1.1.20	AADZ99_RS06495
